# Interpreter Use and Challenges in Primary Care

**DOI:** 10.1001/jamanetworkopen.2026.29269

**Published:** 2026-08-14

**Authors:** Debbie W. Chen, Maya Watanabe, Audrey Fan, Tsu-Yin Wu, Megan R. Haymart, Sarah T. Hawley, Lauren P. Wallner

**Affiliations:** 1 Metabolism, Endocrinology and Diabetes, University of Michigan, Ann Arbor; 2 Center for Biostatistics in AIDS Research, Harvard T.H. Chan School of Public Health, Boston, Massachusetts; 3 Department of General Medicine, University of Michigan, Ann Arbor; 4 Center for Health Disparities Innovations and Studies, Eastern Michigan University, Ypsilanti; 5 Department of Internal Medicine, University of Michigan, Ann Arbor; 6 Ann Arbor VA Center for Clinical Management Research

## Abstract

This survey study investigates use of interpreters and challenges experienced by physicians treating patients with limited English proficiency.

## Introduction

In the US, more than 67 million individuals speak a language other than English.^1,2^ According to US census data, more than 25 million individuals have limited English proficiency (LEP), or speak English less than “very well.”^1^ Studies have consistently found that use of professional medical interpreters for patients with LEP is associated with improved patient outcomes.^3,4,5,6^ However, less is known about interpreter use and challenges in primary care.

## Methods

The University of Michigan institutional review board reviewed this survey study and waived the documentation of informed consent, with survey completion indicating consent. This study followed the AAPOR reporting guideline.

In October 2024 to April 2025, we randomly sampled 1100 internal and family medicine physicians from the American Medical Association Masterfile. Of 753 eligible outpatient primary care physicians (PCPs), 267 PCPs responded (35.5% response rate) (eFigure in Supplement 1). Among these, 230 PCPs who reported having patients with LEP in their panel were included in the analytic cohort.

Physicians answered questions about their care of patients with LEP.^7,8^ Physicians rated how often they used 3 types of professional interpreters (in-person, telephone, and audiovisual) and 3 types of ad hoc interpreters (bilingual family, clinical staff, and administrative staff) to facilitate communication, with responses dichotomized as *regular* (always or often) or *limited use* (sometimes, rarely, or never).^8^ Physicians were asked to rate on a 5-point Likert scale (always, often, sometimes, rarely, or never) how often specific factors affect patient care. Physicians rated their confidence in their ability to engage in treatment decision-making with patients with LEP, with responses dichotomized as *very* confident or anything *less than very* (quite, somewhat, a little, or not at all) confident.

Physician and practice characteristics were summarized, with unadjusted and adjusted logistic regression models used to estimate odds of interpreter use. All statistical analyses were performed using complete-case analysis and 2-sided hypotheses, with *P* < .05 considered statistically significant. Analyses were performed using R version 4.2.1 (R Project for Statistical Computing).

## Results

Among 230 PCPs (121 self-identified as male [52.6%]; 55 Asian [23.9%], 12 Black [5.2%], 16 Hispanic [7.0%], and 131 non-Hispanic White [57.0%]), most reported a panel of 1% to 25% patients with LEP (186 PCPs [80.9%]) (Table 1). Survey responders were older than nonresponders (mean [SD] age, 56.9 [11.0] vs 54.3 [12.1] years; *P* = .003) but did not differ significantly by gender, specialty, or US census region.

**Table 1.** Physician and Practice Characteristics

Characteristic	No. (%) (N = 230)
PCP characteristics	
Gender	
Male	121 (52.6)
Female	100 (43.5)
Prefer not to say	2 (0.9)
No response	7 (3.0)
Race and ethnicity^a^	
African-American or Black	12 (5.2)
Asian	55 (23.9)
Hispanic	16 (7.0)
Non-Hispanic White	131 (57.0)
Other	15 (6.5)
No response	1 (0.4)
Specialty	
Family medicine	129 (56.1)
Internal medicine	88 (38.3)
Other^b^	8 (3.5)
No response	5 (2.2)
Time in clinical practice, y	
≤10	28 (12.2)
11-20	59 (25.7)
21-30	87 (37.8)
>30	48 (20.9)
No response^c^	8 (3.5)
Practice characteristics	
Setting	
Private practice	79 (34.3)
Large medical group or staff-model HMO	72 (31.3)
Academic medical center	24 (10.4)
Community health clinic	16 (7.0)
Federally qualified health center	15 (6.5)
Other^d^	18 (7.8)
No response	6 (2.6)
Individuals with LEP in physician patient panel, %^e^	
1-25	186 (80.9)
26-50	21 (9.1)
51-75	9 (3.9)
76-99	11 (4.8)
No response	3 (1.3)
Physician speaks with patients in non-English language	
Yes^f^	91 (39.6)
No	136 (59.1)
No response	3 (1.3)

Abbreviations: HMO, health maintenance organization; LEP, limited English proficiency; PCP, primary care physician.

^a^ Physicians were asked to check all options that best described their race and ethnicity (American Indian or Alaska Native, Asian, Black or African American, Hispanic or Latino, Middle Eastern or North African, Native Hawaiian or Pacific Islander, or White). Each respondent was analyzed as having a single race or ethnicity category. Individuals who self-identified as Hispanic were categorized as Hispanic regardless of any other racial identification. Other race or ethnicity includes American Indian or Alaska Native, Middle Eastern or North African, Native Hawaiian or Pacific Islander, and multiracial. Self-reported data on race and ethnicity were collected as part of the demographic information to help describe survey respondents and understand the composition of the study cohort.

^b^ Other specialty includes physicians who selected *family medicine* or *internal medicine* along with another specialty (*geriatrics *or *other*), *other*, or *geriatrics*.

^c^ Includes 7 physicians who did not provide a response and 1 physician who indicated “more than 20 years” since this latter response could not be precisely categorized as either *21 to 30 years* or *more than 30 years*.

^d^ Other practice setting includes Veterans Affairs medical center, multistate value-based clinic, state-qualified health clinic, and rural health clinic.

^e^ LEP was defined in the survey as applying to individuals whose primary language is not English and who have a limited ability to read, speak, write, or understand the English language.

^f^ Among 91 PCPs who reported speaking with patients in a language other than English, the 3 most commonly spoken languages were Spanish (48 PCPs), Urdu (6 PCPs), and Chinese (6 PCPs). Physicians also rated their proficiency in the identified non-English language using 4 response options (excellent, good, fair, and poor) that were informed by an adapted version of the Interagency Language Roundtable scale.^7^ Most physicians (48 PCPs [52.7%]) rated their proficiency as excellent, whereas 21 (23.1%) reported good and 22 (24.2%) reported fair or poor.

Half (116 PCPs [50.4%]) regularly used at least 1 type of professional medical interpreter when communicating with patients with LEP, while 122 (53.0%) regularly used at least 1 type of ad hoc interpreter. Table 2 shows estimated odds of regularly using professional and ad hoc interpreters, with and without adjustment for physician and practice characteristics. Compared with PCPs in private practice, those at academic centers and large medical group or staff-model health maintenance organizations had higher odds of regularly using professional interpreters (odds ratio [OR], 4.78 [95% CI, 1.49-15.28] and 6.34 [95% CI, 2.79-14.39], respectively) and lower odds of regularly using ad hoc interpreters (OR, 0.22 [95% CI, 0.07-0.64] and 0.38 [95% CI, 0.18-0.80], respectively). Compared with PCPs with 10 or fewer years in practice, those with more years in practice had lower odds of regularly using professional interpreters (21-30 years: OR, 0.15 [95% CI, 0.04-0.63]; >30 years: OR, 0.08 [95% CI, 0.02-0.37]) and higher odds of regularly using ad hoc interpreters (11-20 years: OR, 6.50 [95% CI, 1.95-18.81]; 21-30 years: OR, 3.24 [95% CI, 1.10-9.54]; >30 years: OR, 4.38 [95% CI, 1.34-14.26]).

**Table 2.** Factors Associated With Regular Use of Professional Medical and Ad Hoc Interpreters

Factor	Interpreter use^a^			Regular use of ≥1 type of interpreter, OR (95% CI)	
	Total, No.	Regular, No. (%)	Limited, No. %)	Unadjusted	Adjusted
**Professional medical interpreter (in-person, telephone, or audiovisual)**					
Practice setting					
Private practice	74	21 (28.4)	53 (71.6)	1 [Reference]	1 [Reference]
Academic medical center	22	14 (63.6)	8 (36.4)	4.50 (1.65-12.29)	4.78 (1.49-15.28)
Large medical group or staff-model HMO	68	46 (67.6)	22 (32.4)	5.49 (2.69-11.22)	6.34 (2.79-14.39)
Other^b^	48	32 (66.7)	16 (33.3)	5.14 (2.35-11.26)	4.56 (1.85-11.23)
Specialty					
Family medicine	125	76 (60.8)	49 (39.2)	1 [Reference]	1 [Reference]
Internal medicine	82	32 (39.0)	50 (61.0)	0.42 (0.24-0.74)	0.52 (0.26-1.03)
Time in practice, y					
≤10	28	24 (85.7)	4 (14.3)	1 [Reference]	1 [Reference]
11-20	55	35 (63.6)	20 (36.4)	0.29 (0.09-0.96)	0.24 (0.06-1.04)
21-30	84	41 (48.8)	43 (51.2)	0.16 (0.05-0.49)	0.15 (0.04-0.63)
>30	44	13 (29.5)	31 (70.5)	0.07 (0.02-0.24)	0.08 (0.02-0.37)
Gender					
Male	116	53 (45.7)	63 (54.3)	1 [Reference]	1 [Reference]
Female	94	59 (62.8)	35 (37.2)	2.04 (1.17-3.54)	2.03 (1.03-4.01)
**Ad hoc interpreter (bilingual family member, clinical staff, or administrative staff)**					
Practice setting					
Private practice	76	51 (67.1)	25 (32.9)	1 [Reference]	1 [Reference]
Academic medical center	22	8 (36.4)	14 (63.6)	0.27 (0.10-0.74)	0.22 (0.07-0.64)
Large medical group or staff-model HMO	69	31 (44.9)	38 (55.1)	0.39 (0.20-0.77)	0.38 (0.18-0.80)
Other^b^	47	27 (57.4)	20 (42.6)	0.65 (0.31-1.37)	0.73 (0.31-1.69)
Specialty					
Family medicine	121	63 (52.1)	58 (47.9)	1 [Reference]	1 [Reference]
Internal medicine	86	52 (60.5)	34 (39.5)	1.39 (0.79-2.43)	1.25 (0.67-2.32)
Time in practice, y					
≤10	27	8 (29.6)	19 (70.4)	1 [Reference]	1 [Reference]
11-20	53	34 (64.2)	19 (35.8)	4.25 (1.57-11.54)	6.05 (1.95-18.81)
21-30	86	46 (53.5)	40 (46.5)	2.79 (1.10-7.05)	3.24 (1.10-9.54)
>30	46	28 (60.9)	18 (39.1)	3.69 (1.34-10.21)	4.38 (1.34-14.26)
Gender					
Male	118	63 (53.4)	55 (46.6)	1 [Reference]	1 [Reference]
Female	93	53 (57.0)	40 (43.0)	1.14 (0.66-1.97)	1.16 (0.63-2.16)

Abbreviations: HMO, health maintenance organization; OR, odds ratio.

^a^ Physicians were asked to rate how often they used 3 types of professional interpreters (in-person, telephone, and audiovisual) and 3 types of ad hoc interpreters (bilingual family, clinical staff, and administrative staff) to facilitate communication, with responses dichotomized as regular use (always or often) or limited use (sometimes, rarely, or never). All statistical analyses were performed using complete-case analysis. Percentages are out of totals per row.

^b^ Other practice setting includes Veterans Affairs medical center, multistate value-based clinic, state-qualified health clinic, and rural health clinic.

Most PCPs endorsed logistical barriers to interpreter access (ie, limited access to any of 3 types of professional interpreters, insufficient clinic visit time, or inadequate time to arrange for professional interpreters; 180 PCPs [78.3%]) and patient interpretation preferences (ie, declining use of professional interpreters or preferring that family or friends interpret; 179 PCPs [77.8%]) affecting their care of patients with LEP at least *sometimes*. Notably, 69 PCPs (30.0%) endorsed concerns about the accuracy of professional language interpretation impacting their care delivery at least sometimes.

Nearly three-quarters of PCPs (163 [70.9%]) reported being less than very confident in their ability to engage in treatment decision-making with patients with LEP. These PCPs, vs 64 very confident PCPs (27.8%), were less likely to speak with patients in a non-English language (55 [33.7%] vs 36 [56.3%]; *P* = .002) and more likely to endorse the following factors as frequently affecting their patient care at least *sometimes*: concerns about accuracy of professional interpretation (59 [37.8%] vs 10 [16.9%]; *P* = .006), limited access to in-person (94 [61.0%] vs 23 [40.4%]; *P* = .01) and telephone (56 [36.8%] vs 7 [12.7%]; *P* = .002) interpreters, inadequate time to arrange for professional interpreters (77 [49.7%] vs 17 [28.3%]; *P* = .007), inadequate clinic time (118 [74.7%] vs 35 [57.4%]; *P* = .02), and patients preferring that family or friends interpret (129 [83.2%] vs 39 [65.0%]; *P* = .007).

## Discussion

This survey study highlights challenges faced by PCPs in delivering care to patients with LEP. Most surveyed physicians endorsed logistical challenges to accessing professional interpreters, and half regularly used untrained ad hoc interpreters, practices that can increase the risk for patient harm.^3^

Our study has limitations, including the cross-sectional design, which precludes causal inference; modest response rate; self-reported data with potential for social desirability bias; and limited generalizability outside primary care. However, by surveying PCPs across diverse practice settings, we found data underscoring the need for interventions that address system-level barriers to interpreter access in primary care.^9,10^ Such efforts are essential to improving care for linguistically marginalized patients.

## Notes

Supplement 1. **eFigure.** Cohort Flowchart

Supplement 2.
Data Sharing Statement
