# Supplementary material for: Interpreter Use and Challenges in Primary Care
Source: JAMA Netw Open. 2026 Aug 14;9(8):e2629269. doi: 10.1001/jamanetworkopen.2026.29269 (PMC13476832; doi:10.1001/jamanetworkopen.2026.29269)
Supplement: Supplementary file 1 [file jamanetwopen-e2629269-s001.pdf]

Supplemental Online Content

Chen DW, Watanabe M, Fan A, et al. Interpreter use and challenges in primary care. *JAMA Netw Open*. 2026;9(8):e2629269. doi:10.1001/jamanetworkopen.2026.29269

eFigure. Cohort Flowchart

This supplemental material has been provided by the authors to give readers additional information about their work.

© 2026 Chen DW et al. *JAMA Network Open*.

eFigure. Cohort Flowchart

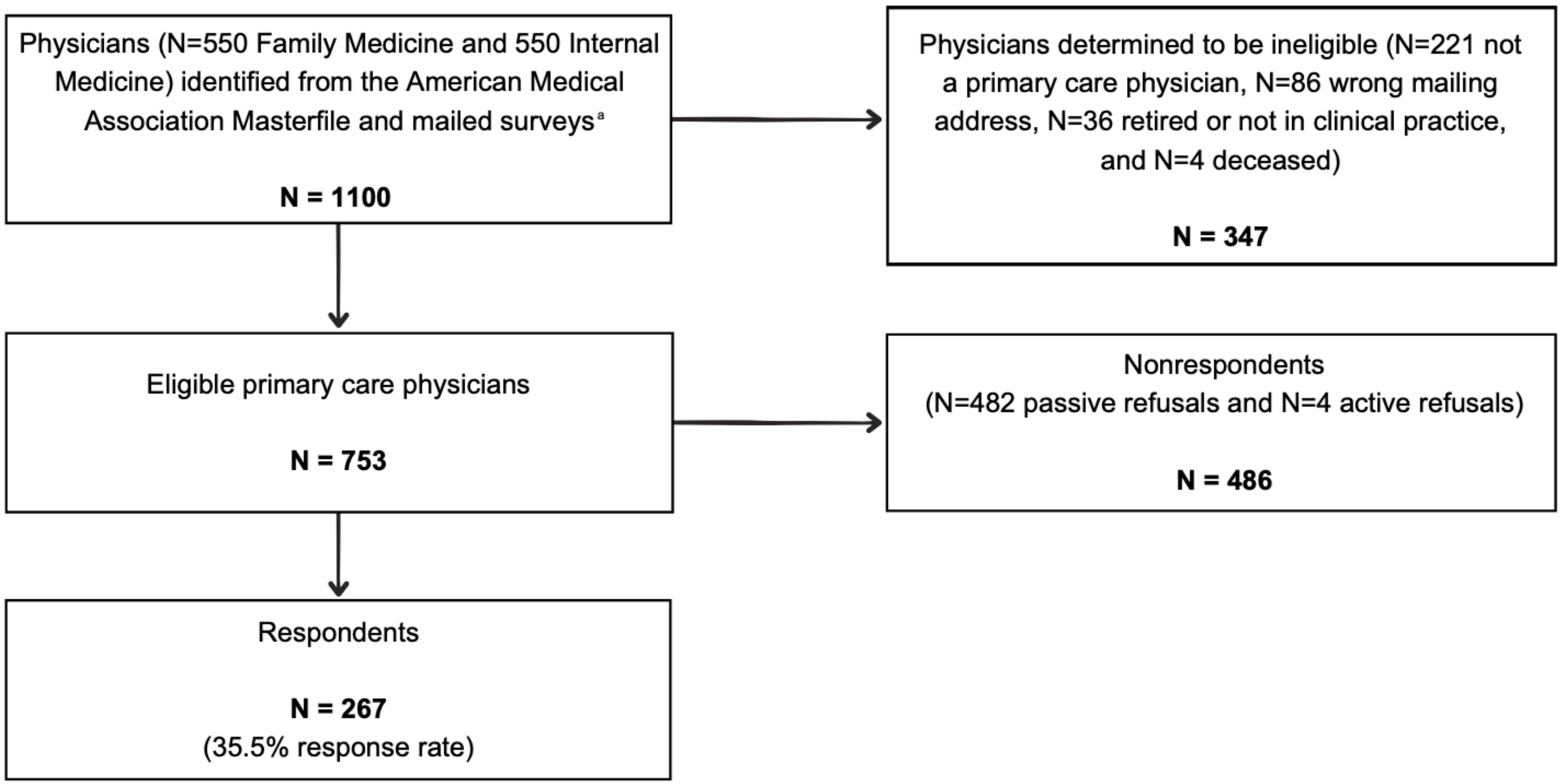

[a] The modified Dillman method of survey administration was used. Online profiles of non-responders were searched to identify those who were deceased, retired, or not in clinical practice.

© 2026 Chen DW et al. *JAMA Network Open*.
