# Supplementary material for: Interpreter Use and Challenges in Primary Care
Source: JAMA Netw Open. 2026 Aug 14;9(8):e2629269. doi: 10.1001/jamanetworkopen.2026.29269 (PMC13476832; doi:10.1001/jamanetworkopen.2026.29269)
Supplement: Supplementary file 2 [file jamanetwopen-e2629269-s002.pdf]

Data Sharing Statement

Chen. Interpreter Use and Challenges in Primary Care. JAMA Netw Open. Published online August 14, 2026. doi:10.1001/jamanetworkopen.2026.29269

Data
Data available: Yes
Data types: Deidentified participant data
How to access data: Requests should be directed to corresponding author (Debbie Chen, MD at chendeb@med.umich.edu). To gain access, data requestors will need to sign a data access agreement.
When available: With publication

Supporting Documents
Document types: None

Additional Information
Who can access the data: Summary-level data may be available from the authors upon reasonable request. Requests should be directed to corresponding author (Debbie Chen, MD at chendeb@med.umich.edu). To gain access, data requestors will need to sign a data access agreement.
Types of analyses: Requests should be directed to corresponding author (Debbie Chen, MD at chendeb@med.umich.edu). To gain access, data requestors will need to sign a data access agreement.
Mechanisms of data availability: Requests should be directed to corresponding author (Debbie Chen, MD at chendeb@med.umich.edu). To gain access, data requestors will need to sign a data access agreement.
